# Rapidly Growing Thyroid Mass in an Immunocompromised Young Male Adult

**DOI:** 10.1155/2013/290843

**Published:** 2013-07-09

**Authors:** Mónica Santiago, José Hernán Martinez, Coromoto Palermo, Carlos Figueroa, Oberto Torres, Rafael Trinidad, Eva Gonzalez, Maria de Lourdes Miranda, Miosotis Garcia, Guillermo Villamarzo

**Affiliations:** ^1^Department of Endocrinology, Metabolism, and Diabetes, San Juan City Hospital, Puerto Rico; ^2^Department of Surgical Pathology and Cytopathology, Hato Rey Pathology Associates Inc., Puerto Rico

## Abstract

We describe a 20-year-old man diagnosed with a myelodysplastic syndrome (MDS), admitted to our hospital due to pancytopenia and fever of undetermined origin after myelosuppression with chemotherapy. Disseminated aspergillosis (DIA) was suspected when he developed skin and lung involvement. A rapidly growing mass was detected on the left neck area, during hospitalization. A thyroid ultrasound reported a 3.7 × 2.5 × 2.9 cm oval heterogeneous structure, suggestive of an abscess versus a hematoma. Fine needle aspiration of the thyroid revealed invasion of aspergillosis. Fungal thyroiditis is a rare occurrence. Thyroid fungal infection is difficult to diagnose; for this reason it is rarely diagnosed antemortem. To our knowledge, this is the 10th case reported in the literature in an adult where the diagnosis of fungal invasion to the thyroid was able to be corroborated antemortem by fine needle aspiration biopsy.

## 1. Introduction

Thyroiditis by fungal organisms is infrequent. Most cases have been reported on immunocompromised patients, such as those with organ transplant, leukemias, receiving certain types of chemotherapy, subjects with human immunodeficiency virus, and others types of immunosuppression. This uncommon site of infections could be explained by the unique features of the thyroid gland [1], that includes protective mechanisms, such as rich blood supply, separation of the neck by fascial planes, high iodine content, and a fibrous capsule [2]. Among the different types of fungal thyroiditis, *Aspergillus * spp. are the predominant causative fungus for thyroiditis and asymptomatic thyroid infiltration [2–4]. *Aspergillus* thyroiditis (AT) has primarily been a postmortem diagnosis on immunocompromised patients with diagnosed disseminated invasive Aspergillosis (DIA) [5, 6]. Reports of antemortem diagnosis are rare, and for this reason in the literature it is difficult to find reports of surviving patients. Here, we report a case describing AT, in an immunocompromised young adult male with a rapidly growing thyroid mass; diagnosis was able to be performed antemortem by FNA cytology. We also review the epidemiology, clinical manifestations, diagnosis, and outcome of the different AT cases reported in the medical literature published during the years 1980–2012 through a search of the PubMed database. 

## 2. Case

 A 20-year-old man was admitted to our hospital on July 2012. He was diagnosed as having an MDS one month before; by that moment he had received two courses of chemotherapy consisting of azacitidine. Admission to the hospital was due to pancytopenia and fever of undetermined origin. 

**Figure 1:**
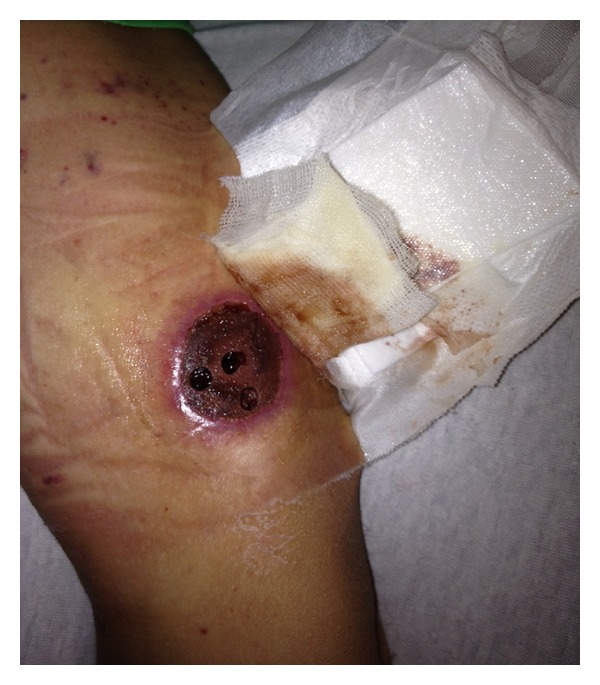
Cutaneous aspergillosis.

**Figure 2:**
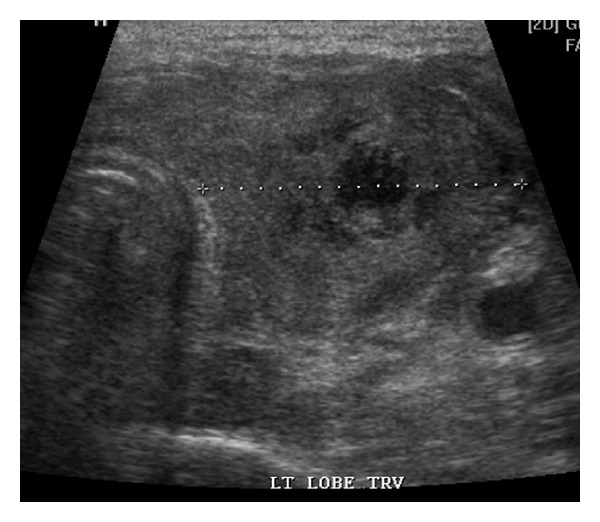
Thyroid sonogram showing a 3.7 × 2.5 × 2.9 cm oval heterogeneous structure, in the left lobe.

 During hospitalization, initial chest X-ray revealed a right perihilar rounded confluent pulmonary opacity, and a subsequent noncontrast-enhanced tomography confirmed the infiltrating nodular lesion measuring 2.5 × 2.3 × 1.9 cm. With these findings and the skin lesions (Figure 1), DIA was suspected. Intravenous liposomal amphotericin B (5 mg/Kg daily) was initiated. One week after hospitalization, a rapidly painless growing mass was detected on the left neck area by the patient. Physical examination revealed a diffusely enlarged nontender palpable mass in the left thyroid lobe. Laboratory data showed normal thyroid function tests (Table 1). A thyroid ultrasound (Figure 2) revealed a 3.7 × 2.5 × 2.9 cm oval heterogeneous structure, suggestive of an abscess versus a hematoma.

**Figure 3:**
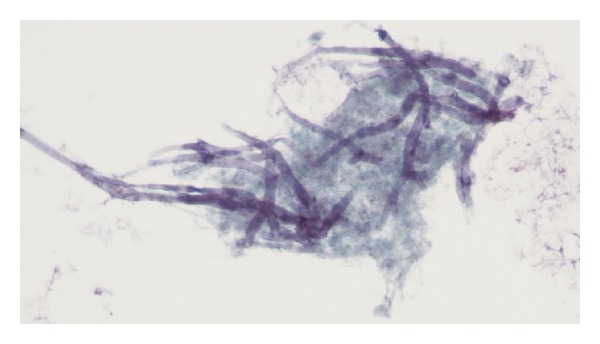
The histological appearance of *Aspergillus* hyphae with septae, branching at 45° (silver methenamine, ×1000).

Microscopy of the thyroid, after fine needle aspiration, revealed infectious thyroiditis with suppurative inflammation and abundant debris. Septate fungal hyphae with branching at acute angles were identified (Figure 3). These findings were consistent with a fungal thyroiditis caused by *Aspergillus* spp. A culture of the aspirated fluid showed no growth. At this moment the patient was switched from previous antifungal to voriconazole (4 mg/Kg IV q 12 hr).

Two weeks after the initiation of voriconazole, he developed wheezing and dyspnea. His respiratory function deteriorated rapidly, needing an endotracheal intubation, and he was further transferred to the intensive care unit, where he finally died.

## 3. Discussion

Fungal pathogens are increasingly encountered on immunocompromised patients. Fungal thyroiditis is a rare occurrence. In the last decade, more than 500 cases of different infectious thyroiditis have been reported, but few cases were fungal, most likely due to the unique features of the thyroid gland, that includes protective mechanisms, such as rich blood supply, separation of the neck by fascial planes, high iodine content, and a fibrous capsule [2]. Although several fungi may infect the thyroid gland [2, 4, 14–18], thyroid fungal infection occurs rarely and is clinically overt in a minority of patients.

In a review of 41 fungal thyroiditis cases published between 1970 and 2005, Goldani et al. [19] found that *Aspergillus* species (spp.) were the most commonly reported cause of fungal thyroid infection. *Candida* spp. were the second most common cause; other fungal etiologies reported include *Cryptococcus neoformans*, *Coccidioides immitis*, *Histoplasma capsulatum*, and *Pseudallescheria boydii,* while *P. jiroveci *is the most common cause of fungal thyroiditis in patients with AIDS, reflecting the high incidence of pneumocystosis in these patients [20]. This observation could suggest that *Aspergillus* spp. have an increased propensity relative to other fungus to infiltrate the thyroid gland or that *Aspergillus *spp. are more likely to cause enough destruction of thyroid tissue to cause symptomatic disease.

We performed a review of the literature of the cases reported with *Aspergillus* thyroiditis in patients over 18 years of age in the medical literature published during 1980–2012 through a search of the PubMed database [4, 7–13, 21–34]. Here, we only report those cases where the diagnosis was able to be corroborated antemortem by fine needle aspiration (FNA) cytology (Table 2). 

The infection by *Aspergillus* spp. is difficult to diagnose; since more than 50% of patients do not exhibit clinical or laboratory manifestations of thyroid dysfunction [5]. Thyroid involvement by *Aspergillus* was found at autopsy as part of disseminated aspergillosis in 11 (46%) of 24 patients without clinical manifestations and laboratory evidence of thyroid dysfunction. Nineteen (79%) of the 24 patients with AT died, most likely due to the underlying immunosuppression associated with disseminated fungal infection and the delay in diagnosis and treatment. Although involvement of the thyroid gland has been detected at autopsy in patients with disseminated fungal disease, there are few reports that have detected the infection by fine needle aspiration (FNA) cytology antemortem (Table 2). To our knowledge, this is the 10th case reported where FNA biopsy plays a major role in diagnosing this entity.

Finally, *Aspergillus* thyroiditis is difficult to diagnose without biopsy, but should be considered in the differential diagnosis of any thyroid nodule, mass, or abscess, particularly in patients with conditions causing immunodeficiency. Besides, the survival of patients with invasive aspergillosis depends on the early diagnosis and prompt initiation of therapeutic measures. In conclusion, we report a case describing *Aspergillus* thyroiditis, in a immunocompromised young adult male with a rapidly growing thyroid mass; diagnosis was able to be performed antemortem by FNA cytology. 

## Figures and Tables

**Table 1:** Results of the patient's laboratory tests.

Test	Result	Normal Range (unit)
White blood cell count	2,000	5,000–10,000 (/mm^3^)
Neutrophils	21	55–75 (%)
Lymphocytes	70	20–44 (%)
Monocytes	11.6	2–8 (%)
Haemoglobin	6.4	12–16 (g/dL)
Haematocrit	18.9	37.0–47.0 (%)
Patelet count	17,000	150,000–450,000 (/mm^3^)
C-reactive protein	374.9	0.1–5.0 (mg/L)
Total thyroxine (T4)	6.36	4.5–12.0 (*μ*g/dL)
Thyroid stimulating		
hormone (TSH)	1.07	0.3–4.0 (mIU/L)

**Table 2:** Review of the medical literature describing cases of the *Aspergillus* thyroiditis diagnosed antermortem by fine needle aspiration (FNA) cytology in patient over 18 years old.

Reference	Year	Age (years/sex)	Comorbidity	Thyroid function	Treatment of thyroid dysfunction	Outcome
Solary et al. [7]	1987	43/F	Renal transplant recipient	Euthyroid	Not reported	Deceased

Torres et al. [8]	1999	24/F	Systemic erythematous disease, end stage renal disease	Hyperthyroid	Not reported	Deceased

Ayala et al. [9]	2001	31/F	Acquired immunodeficiency syndrome	Hyperthyroid	Atenolol	Deceased

Jang et al. [10]	2004	49/F	Acute lymphoblastic leukemia	Hyperthyroid	Not reported	Survived

Sion et al. [4]	2004	46/M	Renal transplant recipient	Euthyroid (↓ TSH)	Not reported	Deceased

Sion et al. [4]	2004	49/?	Renal transplant recipient	Not reported	Hemithyroidectomy	Deceased

Elzi et al. [11]	2005	62/M	Liver transplant recipient	Not reported	Total thyroidectomy	Deceased

Matsui et al. [12]	2006	56/M	Renal transplant recipient	Not reported	Not reported	Survived

Guetgemann et al. [13]	2006	30/F	Renal transplant recipient	Hyperthyroid	NSAIDS beta blockers	Survived

Santiago et al. Current Publication	2013	20/M	Myelodysplastic syndrome	Euthyroid	Not required	Deceased
